# Can Cytoreductive Nephrectomy Improve Outcomes of Nivolumab Treatment in Patients with Metastatic Clear-Cell Renal Carcinoma?

**DOI:** 10.3390/curroncol31090384

**Published:** 2024-09-04

**Authors:** Birol Ocak, Ahmet Bilgehan Sahin, Ismail Ertürk, Mustafa Korkmaz, Dilek Erdem, Umut Cakıroglu, Mustafa Karaca, Ahmet Dirican, Omer Fatih Olmez, Sabin Goktas Aydın, Ali Gökyer, Ahmet Kücükarda, Ahmet Gülmez, Perran Fulden Yumuk, Nazim Can Demircan, Abdilkerim Oyman, Teoman Sakalar, Fatih Karatas, Hacer Demir, Ayse Irem Yasin, Adem Deligonul, Bahar Dakiki, Mehmet Refik Goktug, Okan Avcı, Seher Yildiz Tacar, Nazım Serdar Turhal, Gülhan Ipek Deniz, Turgut Kacan, Erdem Cubukcu, Türkkan Evrensel

**Affiliations:** 1 Department of Medical Oncology, Bursa Yuksek Ihtisas Training and Research Hospital, University of Health Sciences, Bursa 16350, Turkey; turgut.kacan@atlas.edu.tr; 2 Department of Medical Oncology, School of Medicine, Bursa Uludag University, Bursa 16059, Turkey; absahin@uludag.edu.tr (A.B.S.); ademd@uludag.edu.tr (A.D.); bahardakiki@uludag.edu.tr (B.D.); mrgoktug@uludag.edu.tr (M.R.G.); erdemcubukcu@uludag.edu.tr (E.C.); evrensel@uludag.edu.tr (T.E.); 3 Department of Medical Oncology, Gulhane School of Medicine, University of Health Sciences, Ankara 06018, Turkey; ismail.erturk@sbu.edu.tr; 4 Department of Medical Oncology, School of Medicine, Necmettin Erbakan University, Konya 42090, Turkey; dr.musstafa@gmail.com; 5 Department of Medical Oncology, VM Medical Park Samsun Hospital, Samsun 55200, Turkey; dilek.erdem@medicalpark.com.tr; 6 Department of Medical Oncology, Van Training and Research Hospital, University of Health Sciences, Van 65300, Turkey; umutcakiroglu@yyu.edu.tr; 7 Department of Medical Oncology, Antalya Training and Research Hospital, University of Health Sciences, Antalya 07100, Turkey; mustafakaraca@akdeniz.edu.tr; 8 Department of Medical Oncology, School of Medicine, Celal Bayar University, Manisa 45140, Turkey; ahmet.dirican@ieu.edu.tr; 9 Department of Medical Oncology, Medipol University Hospital, Istanbul 34810, Turkey; ofolmez@medipol.edu.tr (O.F.O.); sabin.goktasaydin@saglik.gov.tr (S.G.A.); 10 Department of Medical Oncology, Department of Internal Medicine, School of Medicine, Trakya University, Edirne 22130, Turkey; aligkyer@trakya.edu.tr (A.G.); ahmetkucukarda@trakya.edu.tr (A.K.); 11 Department of Medical Oncology, School of Medicine, Inonu University, Malatya 44280, Turkey; ahmetgulmez@baskent.edu.tr; 12 Department of Medical Oncology, School of Medicine, Marmara University, Istanbul 34854, Turkey; fyumuk@ku.edu.tr (P.F.Y.); nazim.demircan@marmara.edu.tr (N.C.D.); 13 Department of Medical Oncology, Umraniye Training and Research Hospital, University of Health Sciences, Istanbul 34764, Turkey; abdilkerim.oyman@yeniyuzyil.edu.tr; 14 Department of Medical Oncology, Necip Fazıl City Hospital, Kahramanmaraş 46050, Turkey; 15 Department of Medical Oncology, Faculty of Medicine, Karabuk University, Karabuk 78100, Turkey; fatihkaratas@karabuk.edu.tr; 16 Department of Medical Oncology, Afyonkarahisar Health Sciences University, Afyon 03030, Turkey; hacer.demir@afsü.edu.tr; 17 Department of Medical Oncology, Faculty of Medicine, Bezmialem Vakif University, Istanbul 34093, Turkey; ayasin@bezmialem.edu.tr; 18 Department of Medical Oncology, Tekirdağ Namık Kemal University, Tekirdağ 34093, Turkey; oavci@nku.edu.tr (O.A.); sehertcr@gmail.com (S.Y.T.); 19 Department of Medical Oncology, Anadolu Health Center, Kocaeli 2255, Turkey; nturhal@shsny.com; 20 Department of Medical Oncology, Sisli Hamidiye Etfal Training and Research Hospital, University of Health Sciences, Istanbul 34371, Turkey; gulhan.ipek@acibadem.com

**Keywords:** clear-cell renal-cell carcinoma, cytoreductive nephrectomy, nivolumab, survival

## Abstract

**Background:** This study aimed to investigate the effect of cytoreductive nephrectomy (CN) on the survival outcomes of nivolumab used as a subsequent therapy after the failure of at least one anti-vascular endothelial growth factor (VEGF) agent in patients with metastatic clear-cell renal-cell carcinoma (ccRCC). **Methods:** We included 106 de novo metastatic ccRCC patients who received nivolumab after progression on at least one anti-VEGF agent. Multivariate Cox regression analysis was performed to investigate the factors affecting survival in patients receiving nivolumab. **Results:** Of the 106 de novo metastatic ccRCC patients, 83 (78.3%) underwent CN. There were no statistical differences between the two groups in terms of age, gender, Eastern Cooperative Oncology Group (ECOG) score, tumor size, International Metastatic RCC Database Consortium (IMDC) risk group, number of previous treatment lines, first-line anti-VEGF therapy, or metastasis sites (*p* = 0.137, *p* = 0.608, *p* = 0.100, *p* = 0.376, *p* = 0.185, *p* = 0.776, *p* = 0.350, and *p* = 0.608, respectively). The patients who received nivolumab with CN had a longer time to treatment discontinuation (TTD) [14.5 months, 95% confidence interval (CI): 8.6–20.3] than did those without CN 6.7 months (95% CI: 3.9–9.5) (*p* = 0.001). The median overall survival (OS) was 22.7 months (95% CI: 16.1–29.4). The patients with CN had a median OS of 22.9 months (95% CI: 16.3–29.4), while those without CN had a median OS of 8.1 months (95% CI: 5.6–10.5) (*p* = 0.104). In the multivariate analysis, CN [hazard ratio (HR): 0.521; 95% CI: 0.297–0.916; *p* = 0.024] and the IMDC risk score (*p* = 0.011) were statistically significant factors affecting TTD; however, the IMDC risk score (*p* = 0.006) was the only significant factor for overall survival. **Conclusions:** Our study showed that the TTD of nivolumab was longer in metastatic ccRCC patients who underwent cytoreductive nephrectomy.

## 1. Introduction

Renal-cell carcinoma (RCC) of the renal cortex accounts for 80–85% of all primary renal neoplasms [1]. RCC mainly occurs between the sixth and eighth decades of life, with a male predominance of 2:1 [2,3]. According to 2023 Global Cancer Statistics data, RCC is the 14th most common malignancy in both sexes worldwide, and 30% of cases have metastatic disease at diagnosis [4,5]. Partial or radical nephrectomy is the standard treatment option for patients with non-metastatic RCC [6]. However, 20–40% of RCC patients develop metastasis after curative surgery [7,8,9]. Clear-cell carcinoma accounts for 75–85% of RCCs [10]. For several decades, the removal of the primary tumor, called cytoreductive nephrectomy (CN), was the cornerstone of the treatment of newly diagnosed metastatic RCC (mRCC) [11]. However, the effects of CN in patients with metastatic clear-cell RCC (ccRCC) remain controversial, and its impact on treatment options is not well-studied. The CARMENA trial is the only randomized controlled trial investigating the efficacy of nephrectomy in patients with metastatic ccRCC receiving anti-vascular endothelial growth factor (anti-VEGF) therapy [12]. A post hoc analysis of this trial revealed that upfront CN could be performed in patients with low-volume mRCC and a single intermediate International Metastatic RCC Database Consortium (IMDC) risk factor.

In metastatic RCC, previous interferon trials reported that patients receiving interferon-alpha after CN had better survival outcomes than did those without nephrectomy [13,14]. The CheckMate 214 trial revealed that patients who received nivolumab plus ipilimumab had better overall survival (OS) results than did those who received sunitinib among patients with a previous nephrectomy [15]. In the JAVELIN Renal 101 trial, patients who had a previous nephrectomy and received avelumab plus axitinib had statistically significantly longer OS and progression-free survival (PFS) in a subgroup analysis than did those who received sunitinib [16]. In a recent trial performed in 2022, 10 out of 61 mRCC patients treated with nivolumab plus ipilimumab underwent CN, and the authors concluded that CN could be reasonable in a limited number of cases, possibly resulting in curative nephrectomy due to the durable therapeutic effect of immunotherapy [17].

The CHECKMATE 025 trial showed that nivolumab provided an OS advantage over everolimus in advanced-stage patients who had progressed after receiving at least one anti-VEGF agent [18]. In line with the results of this study, international guidelines recommend nivolumab as a subsequent therapy in patients with metastatic ccRCC [19,20]. However, the CHECKMATE 025 trial [18] and international guidelines [19,20] do not provide information about the effect of CN on survival. Our study aimed to investigate the impact of CN on the survival outcomes of nivolumab in patients with metastatic ccRCC who had progressed after receiving at least one anti-VEGF agent.

## 2. Materials and Methods

### 2.1. Study Population and Data Collection

**Figure 1:**
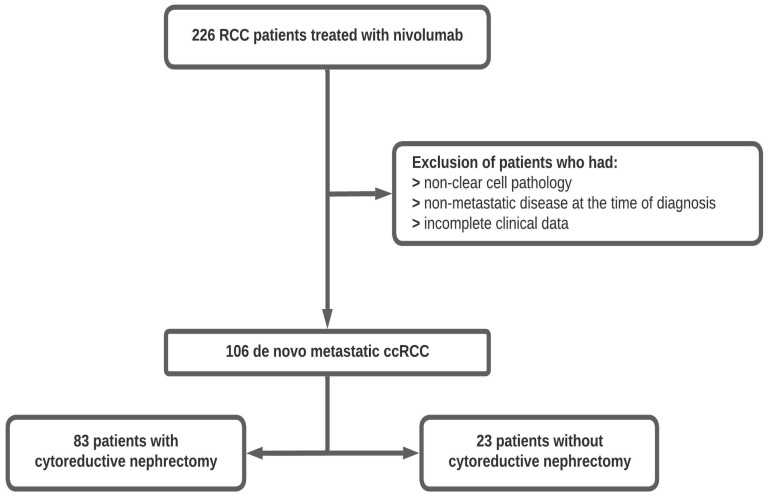
A diagram of the study design. RCC: renal-cell carcinoma, ccRCC: clear-cell renal-cell carcinoma.

This multicenter retrospective study by the Turkish Oncology Group included data obtained from 20 oncology centers. Patients who received nivolumab after progression on at least one anti-VEGF agent for metastatic RCC were included. Patients with autoimmune diseases using glucocorticoids or immunosuppressive agents were excluded from this study. Additionally, among 226 patients, 45 without ccRCC and 75 without metastatic disease at diagnosis were excluded. Among the remaining 106 patients, CN was performed at the diagnosis of metastatic disease in 83 cases. A study flow chart is displayed in Figure 1.

Nivolumab was administered intravenously at a 3 mg/kg dose every two weeks. The time to treatment discontinuation (TTD) refers to clinical deterioration attributed to disease progression that could not be controlled with local ablative treatments or discontinuation due to intolerable side effects. Adverse events and laboratory abnormalities were evaluated using the Common Terminology Criteria for Adverse Events, version 5.0 [21].

The patients were evaluated according to the IMDC risk factors (Karnofsky performance status of <80%, time from diagnosis to treatment of <12 months, hemoglobin below the lower limit of the reference range, serum calcium of >10.0 mg/dL, neutrophil count above the upper limit of the normal range, and platelets above the upper limit of the normal range) [22]. A score of 0 points was accepted as favorable risk, while 1–2 points indicated intermediate risk and 3–6 points indicated poor risk.

### 2.2. Outcomes

Treatment responses were assessed according to the Response Evaluation Criteria in Solid Tumors (version 1.1) [23]. TTD was defined as the time from the date of starting a medication to the date of treatment discontinuation or death. If a clinical benefit continued, nivolumab treatment was continued in the progression, and local ablative therapies were performed. OS was defined as the time from the beginning of nivolumab treatment until death from any cause.

### 2.3. Statistical Analysis

Statistical analyses were performed using SPSS (IBM Corp., released 2017, IBM SPSS Statistics for Windows, Version 25.0, Armonk, NY, USA: IBM Corp.) and MedCalc statistical software (trial version 20.009, MedCalc Software bv, Ostend, Belgium; www.medcalc.org; 2024). Variables were presented as median (minimum–maximum) and frequency values. The normality of the variables was assessed using the Shapiro–Wilk and Kolmogorov–Smirnov tests. The Mann–Whitney U test was employed for quantitative variables, while the chi-square test was used for qualitative variables. Kaplan–Meier analysis was employed for survival rates, and comparisons were made with the log-rank test. Possible factors affecting the TTD and OS were examined using Cox regression analysis. A backward stepwise model was used with parameters with *p*-values below 0.25. A *p*-value of <0.05 was considered statistically significant.

## 3. Results

This study included 106 de novo metastatic ccRCC patients. CN was performed in 83 (78.3%) patients. The clinical characteristics of the patients with and without CN are presented in Table 1. There were no statistical differences between the two groups in terms of age, gender, ECOG score, tumor size, IMDC risk group, number of previous treatment lines, first-line anti-VEGF therapy, or metastasis sites (*p* = 0.137, *p* = 0.608, *p* = 0.100, *p* = 0.376, *p* = 0.185, *p* = 0.776, *p* = 0.350, and *p* = 0.608, respectively).

**Figure 2:**
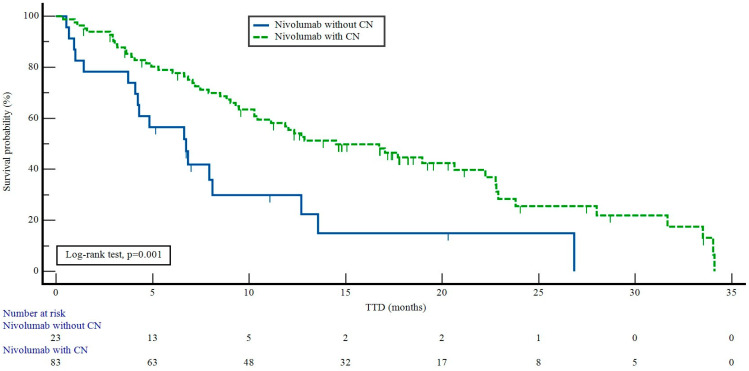
Effect of cytoreductive nephrectomy on time to treatment discontinuation in patients receiving nivolumab. TDT: time to treatment discontinuation, CN: cytoreductive nephrectomy.

The patients who received nivolumab with CN had a median TTD of 14.5 months [95% confidence interval (CI): 8.6–20.3], while the patients who received nivolumab without CN had a median TTD of 6.7 months (95% CI: 3.9–9.5) (*p* = 0.001) (Figure 2).

**Figure 3:**
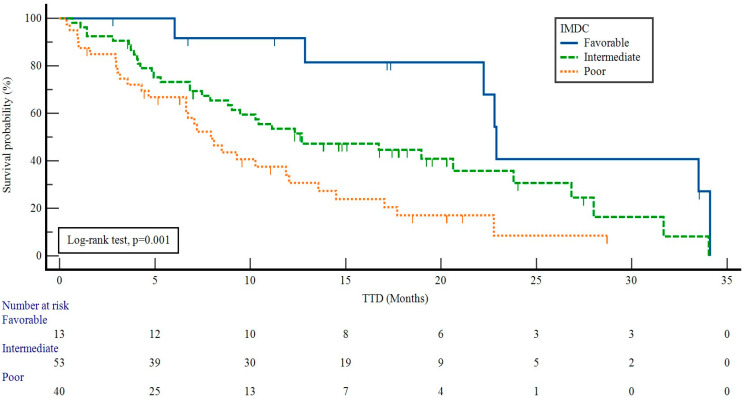
Impact of IMDC risk groups on time to treatment discontinuation in patients receiving nivolumab. IMDC: International Metastatic Renal Cell Carcinoma Database Consortium, TDT: time to treatment discontinuation.

The median TTD values according to the IMDC risk groups were as follows: 22.9 months (95% CI: 22.0–23.7) in patients with favorable risk, 12.7 months (95% CI: 4.7–20.6) in those with intermediate risk, and 7.9 months (95% CI: 5.9–9.9) in those with poor risk (*p* = 0.001) (Figure 3).

The multivariate analysis revealed that CN and the IMDC risk scores were significant independent factors affecting the TTD (with *p*-values of 0.024 and 0.011, respectively) (Table 2).

**Figure 4:**
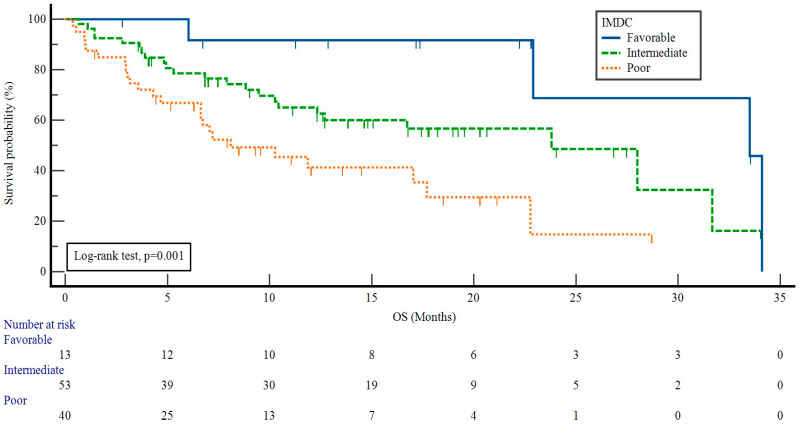
Impact of IMDC risk groups on overall survival in patients receiving nivolumab. IMDC: International Metastatic Renal Cell Carcinoma Database Consortium, OS: overall survival.

The median OS was 22.7 months (95% CI: 16.1–29.4). The patients who received nivolumab with CN had a median OS of 22.9 months (95% CI: 16.3–29.4), while the patients who received nivolumab without CN had a median OS of 8.1 months (95% CI: 5.6–10.5) (*p* = 0.104). The OS values of the patients with favorable, intermediate, and poor IMDC risk were 33.5 months (95% CI: 26.0–40.9), 23.8 months (95% CI: 14.7–32.8), and 8.1 months (95% CI: 2.4–13.7) (*p* = 0.001) (Figure 4).

The IMDC risk groups were the only significant independent factor affecting OS in the multivariate analysis (*p* = 0.006) (Table 3).

Among the patients receiving nivolumab, the objective response rate was 29.2%, and progressive disease was observed in 35 (33.1%) patients. Adverse events associated with nivolumab treatment are listed in Table 4. The most common adverse event was fatigue. No deaths were reported due to adverse events. Nivolumab was discontinued due to pneumonitis in one patient and due to grade 4 hepatitis in another, which were attributed to the treatment.

## 4. Discussion

In the present study, patients who underwent CN were found to have a significantly longer TTD among metastatic ccRCC patients receiving nivolumab after receiving at least one anti-VEGF agent. CN is recommended as a palliative surgery to alleviate complaints such as pain, hematuria, and symptoms arising from paraneoplastic syndromes [24]. In the Checkmate 025 study, 88% of patients underwent nephrectomy, but the effect of CN on survival was not presented [18]. To date, there have been limited studies on the predictive role of CN in the survival of metastatic ccRCC patients receiving nivolumab [25,26,27]. Previous studies investigating the effect of CN on survival with nivolumab are shown in Table 5.

Stellato et al. [25] and Rubuzzi et al. [26] evaluated the effect of nivolumab on survival after therapeutic nephrectomy in non-metastatic disease and CN in the metastatic stage. In these studies, the effects of therapeutic nephrectomy and CN on survival were not presented for subgroups [25,26]. Therefore, it needs to be clarified which subgroup in particular accounts for the survival advantage. Our study responded to these uncertainties by investigating the effect of CN on survival with nivolumab. In studies by Stellato et al. [25], Rubuzzi et al. [26], and Gross et al. [27], patients with clear-cell and non-clear-cell pathologies were evaluated together. In subgroup analyses, clear-cell pathology was not evaluated alone regarding its effect on survival. Our study examined a homogenous group by including patients with clear-cell pathology alone.

Tumor cells secrete various factors that mediate immune suppression. Tumors foster an immune-tolerant microenvironment by increasing interleukin (IL)-6, IL-10, and transforming growth factor beta (TGF-β) secretion. These cytokines promote the infiltration of T regulatory cells, which inhibit the cytotoxic function of T cells [28,29,30]. Additionally, they cause T-cell exhaustion and reduce the ability of T cells to produce cytokines as well as their effector function [29]. Thus, T-cell exhaustion contributes to progressive tumor growth despite CD4+ and CD8+ T cells. BTLA (B- and T-lymphocyte attenuator) is a coinhibitory protein receptor. BTLA down-regulates T-cell function by inhibiting cytokine production [24]. High expression of BTLA is a marker of T-cell exhaustion [29]. Wald et al. [31] investigated changes in the immune systems of patients with non-metastatic ccRCC after nephrectomy. In that study, the circulating levels of BTLA-expressing CD8+ T cells were high in RCC patients before the surgery and rapidly decreased after tumor resection. Therefore, it is thought that T-cell exhaustion and dysfunction may be reversed [31]. In our study, worse survival outcomes during nivolumab treatment in patients without CN may have been associated with systemic immunosuppression caused by the primary tumor.

Studies investigating the impact of CN on immunotherapy responses in metastatic ccRCC patients began in 2001 with interferon (IFN) studies [13,14]. These studies obtained better survival results in metastatic ccRCC patients who received IFN-alpha after CN [13,14]. Fallah et al. [32] showed an OS advantage with immunotherapy combinations in patients with CN compared to those without CN in a Food and Drug Administration pool analysis. In a study by Pignot et al., nephrectomy was performed in patients who had complete responses in metastatic regions after immunotherapy [33]. Although a viable residual tumor was detected in 81.8% of the patients, the authors stated that the disease did not progress in most patients during follow-up (73%) after the primary tumor was removed via nephrectomy [33].

The results of the CARMENA study showed that according to the Memorial Sloan Kettering Cancer Center criteria, sunitinib treatment is more suitable for intermediate- and poor-risk patients than CN, and CN may be considered in the favorable-risk group [12]. In the long-term results of the CARMENA trial, longer overall survival results were obtained in patients with delayed nephrectomy [34]. Stellato et al. [25] found that the IMDC risk groups had statistically significant predictive value for OS and PFS in response to immunotherapy in patients who had previously undergone nephrectomy. These findings were consistent with the results of our study, which indicated that the IMDC risk groups had statistically significant predictive value for OS and TTD in response to nivolumab treatment.

The significant difference in OS in the study by Stellato et al. [25], which was not found in our research, may be due to the exclusion of a patient group with early-stage partial and radical nephrectomy. In addition, since our study included only metastatic ccRCC patients at the time of diagnosis, the proportion of patients with poor IMDC scores was much higher in our study (37.7% vs. 10.1%) than in the study by Stellato et al. [25]. Another reason for the lack of OS benefits may be the inclusion of more patients with high risk.

Tappero et al. found higher survival with CN in metastatic renal-cell carcinoma patients with primary tumors measuring ≤ 4 cm [35]. In our study, no statistically significant effects of tumor size were detected in the multivariate analyses for TTD and OS.

In our study, two patients could not continue the nivolumab treatment due to hepatitis and pneumonitis. The discontinuation rate due to side effects was lower in our study than in the CHECKMATE 025 study (1.8% vs. 8%, respectively) [18]. Our real-world data indicate that nivolumab may be preferred as a subsequent therapy in ccRCC, considering its low toxicity.

To the best of our knowledge, the present study is the first to investigate the impact of CN on TTD in metastatic ccRCC patients receiving nivolumab. We used TTD as a survival outcome, which may have provided a more appropriate evaluation because targeted agents may be continued after progression in cases of clinical benefit [34]. This study’s main limitations are its retrospective nature, the limited number of patients treated with nivolumab without CN, and the inability to use the iRESIST criterion for response evaluation. In addition, surgical techniques and perioperative complications of CN could not be evaluated due to a lack of data.

## 5. Conclusions

Our study showed that the TTD of nivolumab used as a subsequent therapy after at least one anti-VEGF agent was longer in metastatic ccRCC patients who underwent CN. Future randomized prospective studies should be performed to confirm this finding.

## Figures and Tables

**Table 1:** The clinical characteristics of the patients with and without cytoreductive nephrectomy.

		Cytoreductive Nephrectomy (+) *n* = 83	Cytoreductive Nephrectomy (−) *n* = 23	*p*
Age	(Median) (Minimum–Maximum, Years)	59.5 (18.7–78.8)	61.2 (45.6–79.2)	0.137
Gender	Male Female	62 (74.7%) 21 (25.3%)	19 (82.6%) 4 (17.4%)	0.608
ECOG Score	0–1 2	78 (94%) 5 (6%)	19 (82.7%) 4 (17.3%)	0.100
Tumor Size	(Median) (Minimum–Maximum, mm)	90 (20–190)	86 (16–200)	0.376
IMDC Risk Group	Favorable Intermediate Poor	12 (14.5%) 43 (51.8%) 28 (33.7%)	1 (4.3%) 10 (43.5%) 12 (52.2%)	0.185
Previous treatment lines	1 2	64 (77.1%) 19 (22.9)	19 (82.6%) 4 (17.4%)	0.776
First-line anti-VEGF therapy	Sunitinib Pazopanib	51 (61.4%) 32 (38.6%)	11 (47.8%) 12 (52.2%)	0.350
Site of metastasis	Visceral metastasis Non-visceral metastasis	62 (74.7%) 21 (25.3%)	19 (82.6%) 4 (17.4%)	0.608

ECOG: Eastern Cooperative Oncology Group, IMDC: International Metastatic Renal Cell Carcinoma Database Consortium, VEGF: vascular endothelial growth factor.

**Table 2:** Univariate and multivariate Cox regression analyses of the predictors of time to treatment discontinuation.

Factor		Univariate Analysis			Multivariate Analysis		
		HR	95% CI	*p*	HR	95% CI	*p*
Age (years)	≤65 *(R)* vs. >65	0.794	0.434–1.455	*0.456*			
Gender	*Male (R)* vs. *female*	1.084	0.634–1.854	*0.768*			
ECOG score	*0–1 (R)* vs. *2*	1.505	0.683–3.314	*0.310*			
Tumor size (mm)	≤40 *(R)* vs. >40	0.999	0.991–1.006	*0.702*			
Cytoreductive nephrectomy	*No (R)* vs. *yes*	0.419	0.241–0.728	*0.002*	0.521	0.297–0.916	*0.024*
Metastases	*Visceral (R)* vs. *non-visceral*	0.731	0.412–1.298	*0.285*			
IMDC risk group	*Favorable*			*0.002*			*0.011*
	*Intermediate*	2.512	1.029–6.131	*0.043*	2.378	0.970–5.828	*0.058*
	*Poor*	4.561	1.807–11.515	*0.001*	3.917	1.525–10.063	*0.005*
Previous antiangiogenic regimens	*1 (R)* vs. *2*	0.820	0.468–1.437	0.489			

Abbreviations: HR: hazard ratio; CI: confidence interval; R: reference variable; ECOG: Eastern Cooperative Oncology Group; IMDC: International Metastatic Renal Cell Carcinoma Database Consortium.

**Table 3:** Univariate and multivariate Cox regression analyses of the predictors of overall survival.

Factor		Univariate Analysis			Multivariate Analysis		
		HR	95% CI	*p*	HR	95% CI	*p*
Age (years)	≤65 *(R)* vs. >65	0.667	0.313–1.422	*0.295*			
Gender	*Male (R)* vs. *female*	0.883	0.452–1.725	*0.715*			
ECOG score	*0–1 (R)* vs. *2*	0.538	0.130–2.224	*0.392*			
Tumor size (mm)	≤40 *(R)* vs. >40	0.998	0.989–1.007	*0.651*			
Cytoreductive nephrectomy	*No (R)* vs. *yes*	0.571	0.288–1.133	*0.109*	0.742	0.370–1.488	*0.400*
Metastases	*Visceral (R)* vs. *non-visceral*	0.758	0.387–1.487	*0.421*			
IMDC risk group	*Favorable*			*0.003*			*0.006*
	*Intermediate*	3.321	0.951–11.598	*0.060*	3.261	0.932–11.413	*0.064*
	*Poor*	6.903	1.923–24.777	*0.003*	6.496	1.789–23.588	*0.004*
Previous antiangiogenic regimens	*1 (R)* vs. *2*	1.132	0.608–2.109	0.695			

Abbreviations: HR: hazard ratio; CI: confidence interval; R: reference variable; ECOG: Eastern Cooperative Oncology Group; IMDC: International Metastatic Renal Cell Carcinoma Database Consortium.

**Table 4:** Nivolumab treatment-related adverse events.

*Event*	*Grade 1/2* *n (%)*	*Grade 3/4* *n (%)*
Fatigue	24 (22.6%)	2 (1.8%)
Nausea	9 (8.4%)	-
Pruritus	7 (6.6%)	-
Colitis	2 (1.8%)	-
Pneumonitis	1 (0.9%)	1 (0.9%)
Hepatitis	1 (0.9%)	1 (0.9%)
Uveitis	1 (0.9%)	-
Parotitis	1 (0.9%)	-
Hypothyroidism	12 (11.3%)	-

**Table 5:** Studies investigating the effect of cytoreductive nephrectomy on subsequent nivolumab treatment after receiving at least one antiangiogenic agent.

Study	Year	Study Population	Results	Limitations
Stellato et al. [25]	2021	287 patients treated with IO in ≥2 lines	Multivariate analysis revealed that nephrectomy was associated with better OS but not PFS.	Only 33.1% of the patients with nephrectomy had surgery in a metastatic setting. Patients with non-clear-cell pathology were included.
Rubuzzi et al. [26]	2022	571 patients treated with nivolumab in ≥2 lines	Patients who underwent nephrectomy had better OS when they had Meet-URO scores of 1–3.	Patients who underwent nephrectomy in the non-metastatic stage were also included in this study. The ages and distributions of the IMDC groups were statistically different between the nephrectomy and non-nephrectomy groups, and multivariate analysis was not performed. Patients with non-clear-cell pathology were included.
Gross et al. [27]	2023	367 patients treated with IO in any treatment lines	Multivariate analysis revealed that cytoreductive nephrectomy was associated with better OS.	The ages, distributions of IMDC groups, and numbers of patients with CNS and liver metastases were statistically different between the nephrectomy and non-nephrectomy groups. Additional multivariate analysis was not performed for patients receiving IO in ≥2 lines. Patients with non-clear-cell pathology were included.

IO: immune oncology, IMDC: International Metastatic RCC Database Consortium, CNS: central nervous system.

## Data Availability

The data presented in this study are available on request from the corresponding author.
